# Mesenchymal Stem Cell Exosome-Integrated Antibacterial Hydrogels for Nasal Mucosal Injury Treatment

**DOI:** 10.34133/research.0469

**Published:** 2024-09-09

**Authors:** Min Li, Rui Liu, Guopu Chen, Handong Wang, Jinglin Wang, Bin Kong, Chenjie Yu

**Affiliations:** ^1^ Department of Otolaryngology Head and Neck Surgery, Nanjing Drum Tower Hospital, Affiliated Hospital of Medical School, Nanjing University, Nanjing 210008, China.; ^2^ Department of Otolaryngology Head and Neck Surgery, Affiliated Nanjing Drum Tower Hospital Clinical College of Xuzhou Medical University, Nanjing 210008, China.; ^3^ Guangdong Key Laboratory of Biomedical Measurements and Ultrasound Imaging, Department of Biomedical Engineering, School of Medicine, Shenzhen University, Shenzhen, Guangdong 518000, China.; ^4^ Department of Neurosurgery, Health Science Center, The First Affiliated Hospital of Shenzhen University, Shenzhen Second People’s Hospital, Shenzhen, Guangdong 518035, China.

## Abstract

Hydrogels have emerged as appealing prospects for wound healing due to their superior biocompatible qualities. However, the integration of antibacterial active substances into hydrogels for effective wound repair remains challenging. Here, we present a novel double-network hydrogel for nasal mucosal injury repair with antibacterial and self-healing capabilities. This hydrogel is the result of mixing aldehyde polyethylene glycol (PEG) and a carboxymethyl chitosan (CMCS)-based hydrogel with a photocured methylacrylate gelatin (GelMA) hydrogel to envelop mesenchymal stem cell exosomes (MSC-Exos). CMCS is rich in amino groups and facilitates antibacterial repair. Given the dynamically reversible Schiff base connections between the amino group of chitosan and the aldehyde group of modified PEG, the hydrogel can be easily injected into the lesion site because of its excellent injection and shear thinning properties. GelMA introduces an additional network layer for the hydrogel, which enhances its strength and extends the duration of stem cell exosomes on the wound surface. On the basis of these characteristics, we provide evidence that this compound hydrogel can substantially increase cell proliferation and regeneration, inhibit scar hyperplasia, and stimulate angiogenesis in rabbit nasal septum mucosa trauma models. These results suggest that MSC exosome-loaded hydrogels (ME-Gel) have substantial clinical potential for the repair and regeneration of nasal mucosa after surgery or trauma.

## Introduction

The nasal mucosa, a unique location, is highly susceptible to bacterial biofilm formation after injury and infection, which hinders the rate of mucociliary clearance and impedes wound healing [1–5]. Therefore, inhibiting bacterial growth at the lesion site is crucial for wound healing. Traditional drugs and treatment methods, such as antibiotics, intranasal steroid sprays, and polyvinyl acetate foam, are used to prevent postoperative damage to the nasal cavity after surgery. However, none of the above therapies can effectively promote the repair of nasal mucosal injuries and can even cause discomfort due to excessive filling volume [6–8]. Hydrogels provide a moist and gentle repair environment, preventing bacterial attachment while covering wound tissue. In particular, the injectable hydrogel can directly target the loaded drug into the wound via a syringe, extending the residence time of the drug, which is highly promising for treating nasal mucosal injuries [9–13]. However, most reported hydrogels are still supported by traditional medicines with relatively single functions, making it challenging to achieve antibacterial and therapeutic effects [14–16]. Therefore, new dual-effect hydrogels with antibacterial properties and the ability to deliver active substances is urgently needed.

**Fig. 1.**
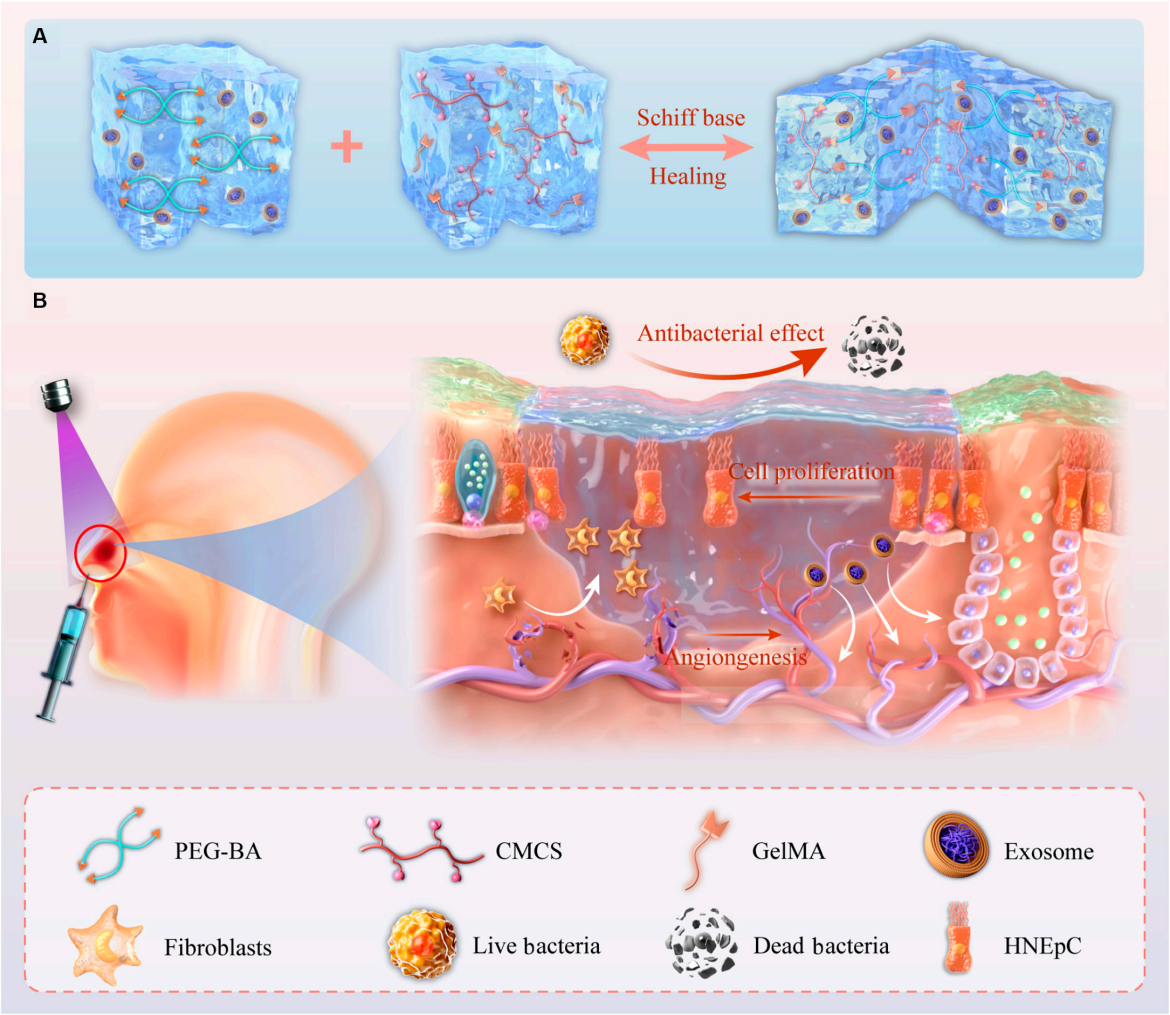
(A and B) Diagrammatic representation of the preparation method of photocured composite hydrogel with MSC-Exos and its application in boosting nasal mucosa injury recovery.

In this work, we propose an antibacterial, self-healing hydrogel loaded with mesenchymal stem cell exosomes (MSC-Exos) to repair nasal mucosal wounds, as shown in Fig. 1. Liquid biopsy, due to minimally invasive detection, which collects biofluids through blood or urine, has emerged as a novel strategy for cancer diagnosis and prediction. Exosomes, recognized as important participants in cellular communication, are rich in multiple bioactive molecules, such as nucleic acids, proteins, and lipids. Extensive evidence suggests that exosomes have great potential as biomarkers in mediating intercellular communication [17–23]. MSC-Exos are extracellular vesicles secreted by MSCs that inherit the repair functions of stem cells, such as promoting angiogenesis and regulating immunity. These vesicles are less immunogenic than stem cells and demonstrate greater biosafety. Additionally, the ability of MSC-Exos to promote cell proliferation, migration, and regeneration makes them promising cell-free therapeutic agents for enhancing tissue regeneration and inhibiting inflammatory diseases [24–31]. To date, chitosan has been widely used in wound repair. As an amphoteric chitosan derivative, carboxymethyl chitosan (CMCS) has excellent antibacterial properties due to its natural cation properties and numerous amino groups. However, the resulting chitosan hydrogels usually form large polymer networks, which cannot maintain the slow release of exosomes, and the poor mechanical properties of this type of hydrogel make it challenging to retain in the nasal mucosa in a complex interfacial environment [32,33]. Thus, the key to developing a new type of hydrogel with dual antibacterial and prorepair properties lies in creating an antibacterial hydrogel that can efficiently encapsulate exosomes [34–45].

Here, we prepared a novel double-network hydrogel containing stem cell exosomes by introducing methylacrylate gelatin (GelMA) into CMCS and a 4-arm benzaldehyde polyethylene glycol (PEGBA) hydrogel. CMCS can be crosslinked with PEGBA to produce self-healing antibacterial hydrogels. The Schiff base connection was created by the aldehyde group, and the amino group imparted excellent injection properties to the hydrogels. The inclusion of GelMA substantially enhanced the mechanical characteristics of the hydrogel and prolonged its action period on nasal mucosal wounds. After considering the benefits mentioned earlier, we conducted in vitro tests to confirm the antimicrobial and prorepair properties of MSC exosome-loaded hydrogels (ME-Gels). The results revealed that the hybrid hydrogel could sustain the release of exosomes while maintaining excellent antibacterial performance. ME-Gel can effectively promote cell proliferation and regeneration. We subsequently demonstrated that the compound hydrogel significantly enhanced cell proliferation and regeneration, inhibited scar hyperplasia, and stimulated angiogenesis in a rabbit nasal septum mucosa trauma model. These results indicate that ME-Gel has excellent potential for clinical application in the antimicrobial repair of nasal mucosal injury.

## Results

### Synthesis and characterization of the hydrogel

**Fig. 2.**
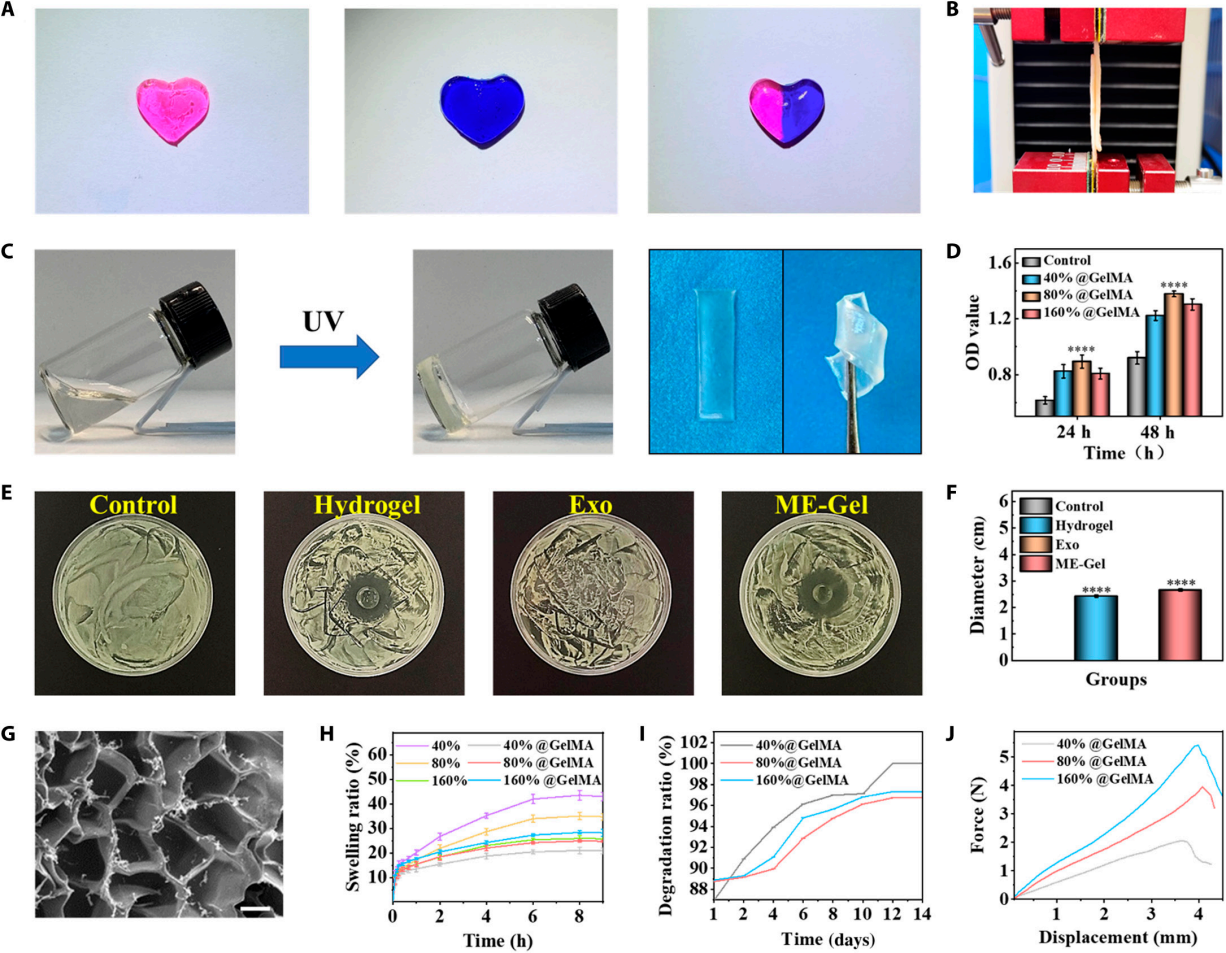
Characterization of the hydrogel. (A) Self-healing traits associated with hydrogels. (B) Testing the adhesion of hydrogels through pigskin. (C) Hydrogels’ photocuring characteristics. (D) Cell viability of nasal mucosal epithelial cells incubated for 1 and 2 d tested by CCK-8 kit (*n* = 3). (E and F) Antibacterial properties of hydrogels. (G) SEM structure of hydrogels with different concentrations. Scale bar, 100 μm. (H) Swelling capacity of different hydrogels. (I) Degradation capacity of hydrogels. (J) Adhesion of hydrogels at different concentrations. *P < 0.05, **P < 0.01, ***P < 0.001, ****P < 0.0001.

Self-healing hydrogels were typically created using CMCS, PEGBA, and GelMA (Fig. 2A and C). This hydrogel possesses excellent adhesion and antimicrobial properties (Fig. 2B, E, and F). We tested the cell activity of the hydrogels at different concentrations. The composite hydrogel with 3% CMCS, 20% PEGBA, and 20% GelMA showed the highest cell activity (Fig. 2D). Scanning electron microscopy (SEM) images (Fig. 2G and Fig. S1) revealed the interconnected pore morphology of the hydrogels. The uniform pore structure of the hydrogel makes it efficient at releasing drugs and retaining water. It is crucial to consider the potential hydrogel degradation with swelling when repairing nasal mucosa injuries. Excessive swelling can negatively affect adhesion and slow the healing process. Our research revealed that the introduction of GelMA inhibited the swelling of CMCS hydrogels (Fig. 2H). When 3 different concentrations of hydrogels were degraded in phosphate-buffered saline (PBS), the hydrogels demonstrated the longest degradation time when the ratio of CMCS to PEGBA was 80% (Fig. 2I). Wet tissue adhesion is crucial when hydrogels are used to treat nasal wounds. We examined the adhesion of the new hydrogel to wet tissues and found that its adhesion ability increased with increasing CMCS concentration (Fig. 2J).

### Preparation and characterization of the MSC-Exos and Exo-loaded hydrogel

**Fig. 3.**
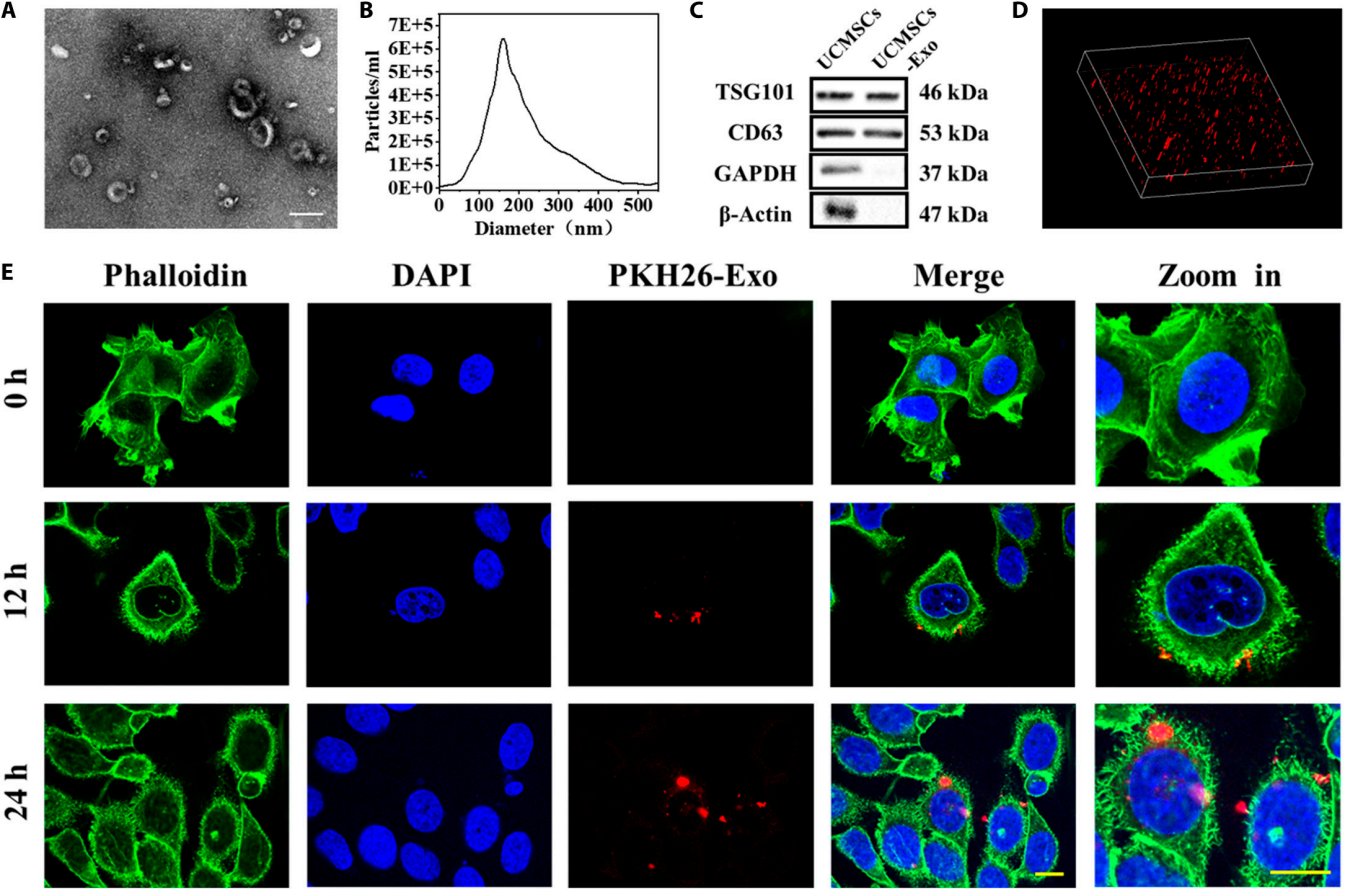
Characteristics of the MSC-Exos. (A) Exos’s morphology assessed by TEM. Scale bar, 200 nm. (B) Allocation of exosome particle sizes determined via NTA. (C) Western blot identification of marker protein molecules. (D) The location of Exos in hydrogel can be observed by 3D immunofluorescence visuals. (E) Immunofluorescence images showed that nasal mucosal epithelial cells phagocytosed PKH26-labeled exosomes at different times. Scale bar, 20 μm.

MSC-Exos were obtained from the cell supernatant via an ultrafiltration kit. The separated Exos were examined using Western blotting, transmission electron microscopy (TEM), and nanoparticle tracking analysis (NTA) (Fig. 3A to C). TEM images revealed the presence of the typical cup-like structure and smooth double-layered structure of Exos. The NTA data revealed that the nanoparticle sizes varied from 40 to 200 nm. The extracted MSC-Exos expressed the exosome markers CD63 and TSG101, whereas the negative markers glyceraldehyde-3-phosphate dehydrogenase (GAPDH) and β-actin were not detected. Confocal microscopy image revealed that Exos were uniformly suspended in the hydrogel (Fig. 3D). Immunofluorescence images confirmed that PKH26-labeled Exos were present around the nuclei of human nasal mucosal epithelial cells, indicating that the released Exos could be effectively phagocytized by human nasal epithelial cells (HNEpCs) (Fig. 3E and Fig. S2).

### Cellular functions of Exo-loaded hydrogels

**Fig. 4.**
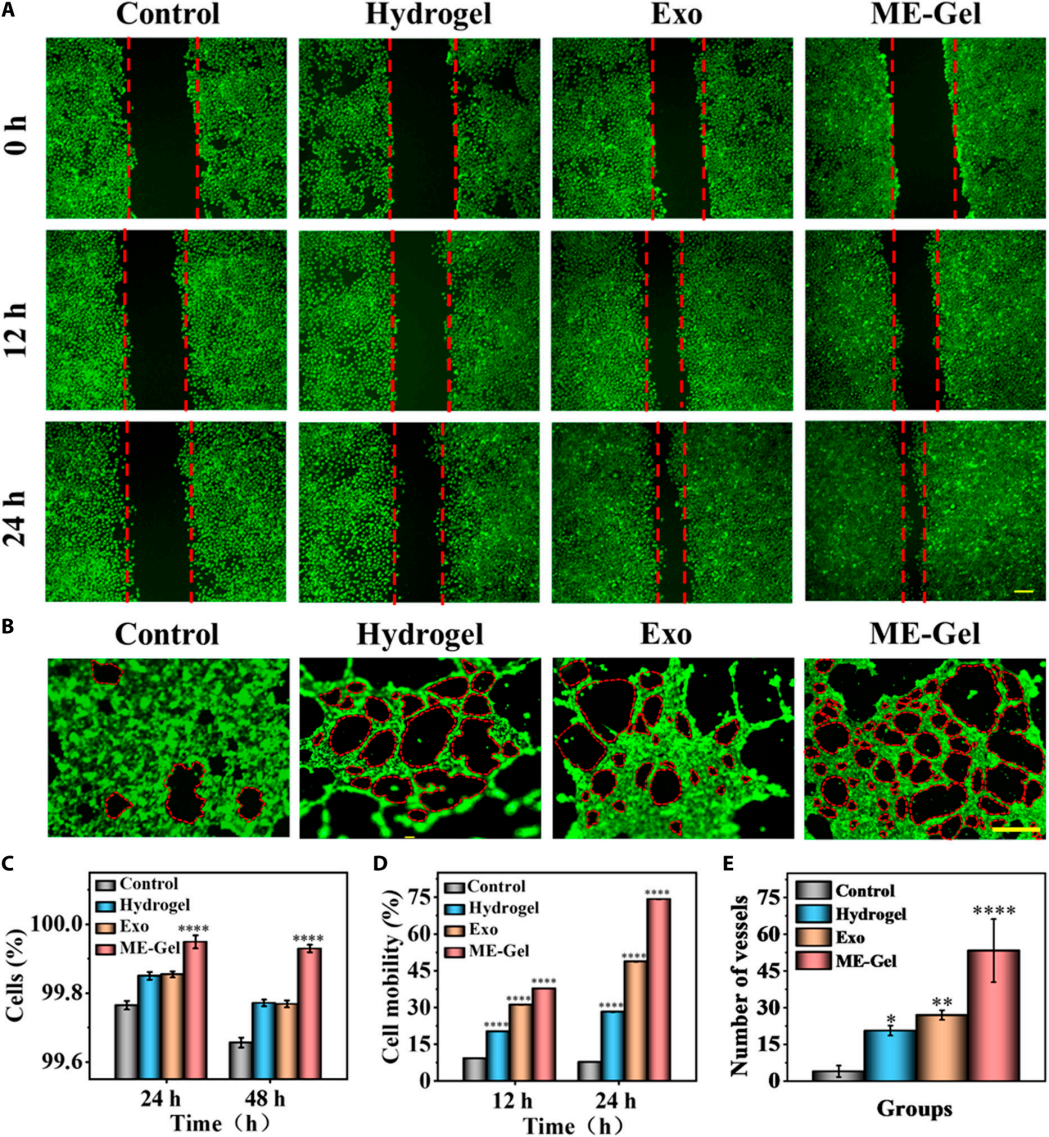
In vitro biocompatibility of the photocured composite hydrogel. (A) Effect of ME-Gel on nasal mucosal epithelial cells evaluated by the scratch wound assays. Scale bar, 100 μm. (B) Effect of hydrogel on angiogenesis. Scale bar, 250 μm. (C) Quantitative analysis of live/dead fluorescence staining. (D) Statistical analysis of fibroblast and HNEpC mobility after different treatments. (E) Quantitative examination of blood vessel counts. *P < 0.05, **P < 0.01, ***P < 0.001, ****P < 0.0001.

The cytocompatibility of hydrogels is essential for the treatment of nasal trauma. Live/dead staining images were acquired during cell cocultivation under various culture conditions to assess the cytocompatibility of the hydrogel, as shown in Fig. S3. The percentage of living cells did not differ significantly between the groups (Fig. 4C). These results indicate that ME-Gel exhibited good cytocompatibility and showed no toxicity to human nasal mucosal epithelial cells. After 24 h of coculture under different conditions, HNEpCs (Fig. 4A and D) and fibroblasts (Figs. S6 and S7) in the ME-Gel group were better able to promote cell migration and accelerate the healing of mucosal wounds. The results were consistent with those of the Transwell test (Figs. S4 and S5). In addition, we investigated ME-Gels’ ability to trigger cell migration and angiogenesis through tubular experiments. The ME-Gel group exhibited a greater ability to promote angiogenesis than the control group (Fig. 4B and E).

### Inhibition of scar hyperplasia and bacterial production by ME-Gel

**Fig. 5.**
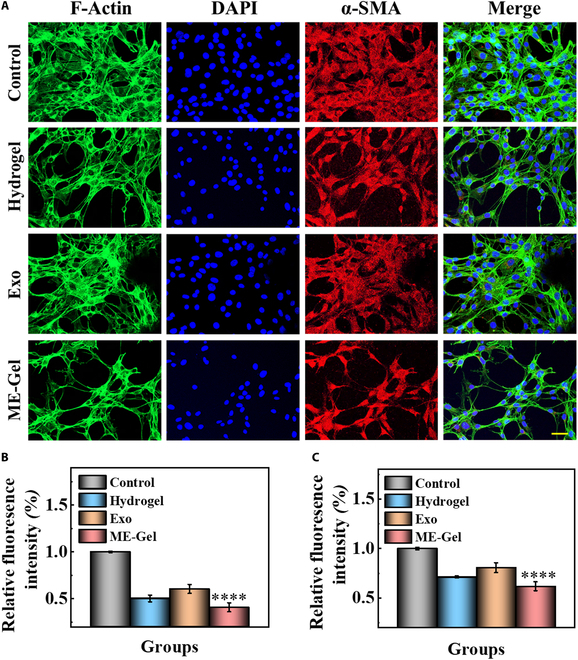
(A) Expression of α-SMA (red) in fibroblasts after coculture with ME-Gel for 1 day. Scale bar, 50 μm. (B) Measuring the mean fluorescence intensity of α-SMA in fibroblasts quantitatively. (C) Quantitative evaluation of integrin’s mean fluorescence intensity in fibroblasts. *P < 0.05, **P < 0.01, ***P < 0.001, ****P < 0.0001.

Following traumatic injury, the creation of scar tissue impedes the ability of nasal epithelial tissue to repair. Therefore, we analyzed the proportion of fibroblasts converted to myofibroblasts and cell adhesion by immunocytochemical staining of α-SMA (smooth muscle actin) (Fig. 5A) and integrin (Fig. S8). After coculture under different conditions for 24 h, all the fibroblast components displayed normal cell morphology and good biocompatibility. However, the diffusion area of the fibroblast cytoskeleton was smaller in the ME-Gel group than in the other 3 groups. The control group demonstrated the largest diffusion area and the highest levels of integrin and α-SMA (Fig. 5B and C). ME-Gel is highly compatible with fibroblasts and can promote their growth and adhesion while preventing excessive proliferation. The results from the bacteriostatic test revealed that there was no bacteriostatic ability within either the control group or the Exo group. In contrast, the hydrogel and Exo groups inhibited the growth of *Staphylococcus aureus* (refer to Fig. 2E and F).

### Exo-loaded hydrogels accelerated nasal wound repair in vivo

**Fig. 6.**
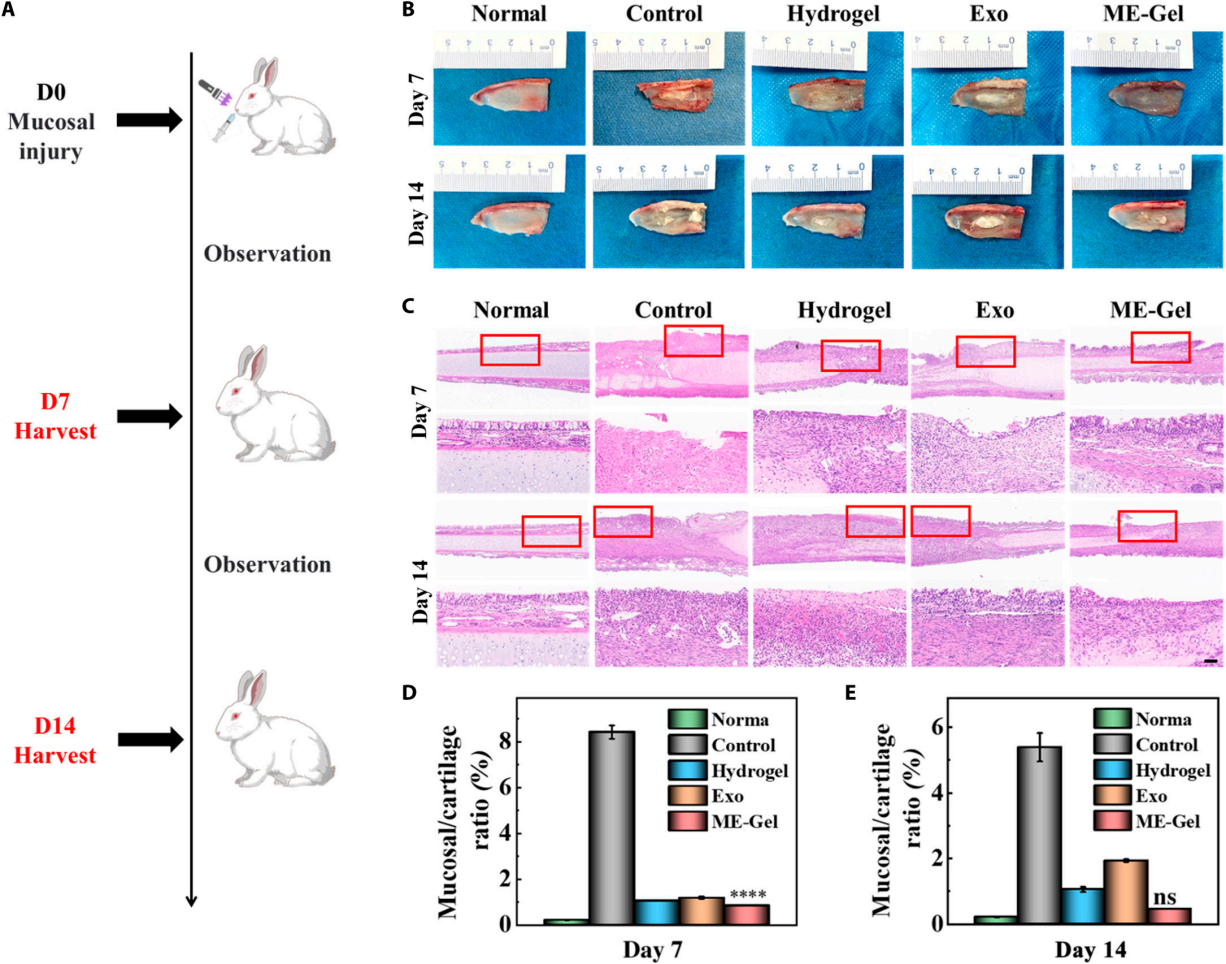
The composite hydrogel’s in vivo nasal mucosa wound healing evaluation. (A) Illustration of an animal experiment’s schematic. (B) Representative images of nasal mucous wounds treated using PBS, hydrogel, MSC-Exos, and ME-Gel on days 7 and 14. (C) Sample photos of the wound conditions on days 7 and 14 after H&E staining. Scale bar, 100 μm. (D) On day 7, the ratio of mucosal thickness on the injured side to the full thickness of the nasal septum. (E) On day 14, the ratio of mucosal thickness on the injured side to the full thickness of the nasal septum. *P < 0.05, **P < 0.01, ***P < 0.001, ****P < 0.0001, not significant (ns).

We subsequently conducted hematoxylin and eosin (H&E) staining experiments on major organs of rabbits to test the toxic effects of each treatment (Fig. S9). As shown in Fig. 6A, the nasal cavity of the rabbits was opened at a width of approximately 15 mm × 5 mm on the mucosal surface of the nasal septum. These rabbits were then divided into 4 groups and treated with PBS, hydrogels, exosomes, or ME-Gel. The nasal incision was sutured after injury to the nasal mucosa. Different wound groups were observed on the 7th and 14th days. The group treated with the Exo-loaded hydrogel in vivo demonstrated significantly better outcomes than the other groups. ME-Gel demonstrated apparent anti-inflammatory and bacteriostatic effects, reduced scar formation, and promoted cell migration and regeneration. These outcomes were consistent with the conclusions drawn from the in vitro tests, as shown in Fig. 6B. The ME-Gel group presented a distinct stratified structure, leading to improved mucosal regeneration. H&E staining revealed no prominent edema, and cilia were regenerated by crawling (Fig. 6C to E). The hydrogel and exosome groups also promoted wound healing of the nasal mucosa to a certain extent. These findings indicate that ME-Gel can potentially promote the regeneration of nasal mucosa wounds.

**Fig. 7.**
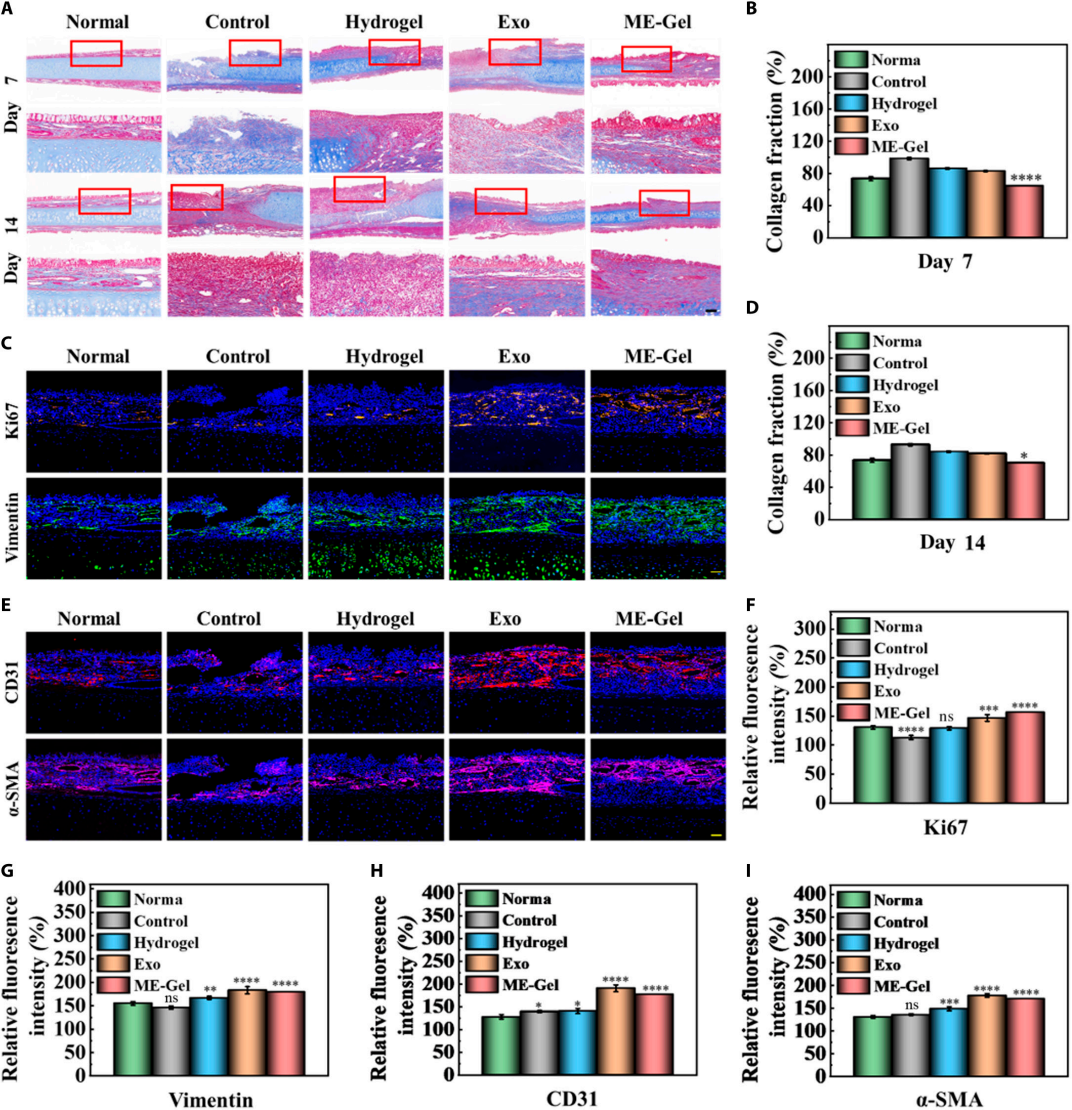
Results of angiogenesis and collagen deposition. (A) Pictures of the nasal mucosa wounds treated by separate groups on days 7 and 14 using Masson staining. Scale bar, 100 μm. (B) Relative content of collagen in nasal septum mucosa by Masson staining on day 7. (C) Ki67 and vimentin immunofluorescence staining pictures taken on day 7 after various groups’ treatments. Scale bar, 100 μm. (D) Relative content of collagen in nasal septum mucosa by Masson staining on day 14. (E) CD31 and α-SMA immunofluorescence staining pictures on day 7 after treatment for various groups. Scale bar, 100 μm. (F) Statistical map of relative fluorescence intensity of Ki67. (G) Statistical map of relative fluorescence intensity of vimentin. (H) Statistical map of relative fluorescence intensity of CD31. (I) Statistical map of relative fluorescence intensity of α-SMA. *P < 0.05, **P < 0.01, ***P < 0.001, ****P < 0.0001.

Masson’s trichrome staining revealed complete regeneration of the full-thickness wound epithelium across the groups, with abundant appendage regeneration in the ME-Gel group. In the hydrogel- or Exos-treated groups, collagen synthesis increased, accompanied by directional alignment, reflecting an improvement in extracellular matrix (ECM) remodeling, which is critical for accelerated wound healing (Fig. 7A, B, and D). We also studied the biological mechanisms involved in the repair process by using immunofluorescence staining to detect Ki67 and the fibroblast marker vimentin. The ME-Gel group exhibited significantly greater cell proliferation than the other groups (Fig. 7C, F, and G). Furthermore, the composite hydrogel provided structural support for the formation of blood vessels, promoting angiogenesis. CD31 and α-SMA staining revealed a marked increase in the density of blood vessels in the wounded granulation tissue, indicating improved wound healing performance in the ME-Gel group (Fig. 7E, H, and I).

## Discussion

Chronic sinusitis is a common disease in otolaryngology. Endoscopic sinus surgery (ESS) is the most commonly used surgical treatment when conservative treatment fails. In the course of ESS surgical treatment, the injury of the nasal mucosa is inevitable and may lead to serious complications, such as nasal adhesions. At present, dilatant sponge is often chosen to compress the surgical area after surgery to stop bleeding, with systemic infusion for anti-inflammation. Traditional expansive sponges used for tamponade often cause nasal obstruction, severe headaches, blurred vision, and other symptoms because of their large size, but also easily cause bacterial reproduction, aggravating nasal infections. In addition, expansive sponges themselves do not promote nasal mucosal healing. Therefore, how to effectively repair the damaged nasal mucosa is still an urgent problem to be solved.

Based on this clinical problem, we are looking forward to a new biodegradable hydrogel with antibacterial and prorepair functions while delivering active substances efficiently. In this study, we propose an antibacterial prorepair hydrogel loaded with MSC-Exos for promoting nasal mucosal wound healing. The hydrogel has good biocompatibility, and after adhering to the nasal wound, it can be cured by ultraviolet (UV) light to extend its self-degradation time, and it does not need to be removed again with instruments like traditional packing materials. In addition, MSC-Exos hydrogel showed remarkable antibacterial effect, which provided a good and suitable healing environment for subsequent mucosal repair. More importantly, the use of MSC-Exos hydrogel can accelerate the healing process of nasal mucosal wounds, facilitate the proliferation of nasal mucosal epithelial cells, promote wound vascular regeneration and collagen deposition, and reduce the generation of local hyperplastic scars.

It is still unclear how MSC-Exos hydrogels involve the specific mechanism underlying epithelial proliferation and remodeling in repairing, and further studies are required to address this issue. Besides, another important limitation of this study is that although we have delayed the degradation time of the hydrogel in vitro as much as possible, the nasal cavity is a complex environment rich in various enzymes, which actually accelerates the degradation of the hydrogel in vivo. After hydrogel degradation, it is difficult to continue to provide a suitable healing environment for the wound, resulting in poor mucosal healing effect. Further research is needed to address this issue.

However, we believe that this MSC-Exos hydrogel can help those patients with nasal mucosal injury to shorten the recovery time, help relieve the suffering, improve the therapeutic effect, thereby improving the quality of life of patients, and provide a new therapeutic idea for promoting the regeneration of clinical nasal mucosal injury, which has great clinical application prospects.

## Materials and Methods

### Materials

Chitosan with a molecular weight of 2 × 10^5^ Da was acquired from Sigma-Aldrich. PEGBA (molecular weight 6,000) was supplied by Ponsure Biotechnology. The cell lines comprising nasal mucosal epithelial cells, fibroblasts, and human umbilical vein endothelial cells (HUVECs) were obtained from the American Type Culture Collection (ATCC). These cell lines were cultured at 37 °C with 5% CO_2_ in Dulbecco’s modified Eagle’s medium (DMEM) (Gibco, USA) supplemented with 10% fetal bovine serum (FBS) and 1% penicillin–streptomycin mixture. The Cell Counting Kit-8 (CCK-8) was obtained from KeyGEN BioTECH Co. Ltd., Nanjing, China. Yeasen Biotechnology Co. Ltd. (Shanghai, China) supplied the Double Stain Kit Calcein-AM/PI. All antibodies—integrin, CD31, α-SMA, Ki67, and vimentin—were purchased from Cell Signaling Technology.

### Cell cultures

MSC-Exos isolated from human cord blood stem cells were used in this study. MSC-Exos were grown in DMEM (Gibco, USA) supplemented with 10% FBS (Corning, USA), 100 U/ml penicillin, and 100 μg/ml streptomycin. The cultures were incubated at 37 °C in a humidified incubator with 5% (v/v) CO_2_. HNEpCs, fibroblasts, and HUVECs from subpassages 3 and 5 were used for the subsequent tests. The fourth and sixth generations of cells were used in the experiments, and the culture medium was changed every 2 to 3 d.

### Synthesis and characterization of the hydrogel

The hydrogels were synthesized through the amalgamation of various solutions. Two solutions were prepared by dissolving the product in the cell culture medium: a 3% (w/v) CMCS solution and a 20% (w/v) PEGBA solution. The gelatin solution (porcine skin gelatin type A, Sigma-Aldrich) was mixed with 20% methacrylic anhydride (Sigma-Aldrich, MO, USA) for 1 h. A white porous GelMA foam was produced by diluting the resulting solution with phosphate buffer, storing it for 5 d, and then freeze-drying it for 1 week. For the preparation of Exo-loaded GelMA hydrogels, 200 μg of foam was combined with 0.05% (w/v) photoinitiator (lithium phenyl-2,4,6-trimethylbenzoylphosphinate, Sigma-Aldrich) in GelMA hydrogel solution. A 3-dimensional (3D) GelMA hydrogel loaded with Exos was created by photocrosslinking the combined material under UV light. The adhesion properties of the samples were assessed by applying them to pigskin surfaces and subjecting the skin samples to twisting and pulling maneuvers.

### PKH26 staining assay

For fluorescent tracking of internalization, the red colorant PKH26 from Sigma-Aldrich (USA) was used to identify the MSC-Exos. The MSC-Exos were incubated with 500 μl of dilution solution C and 4 μl of PKH26 dye solution for 5 min in the dark. Then, 500 μl of 1% bovine serum albumin was added to stop the staining. After fluorescent labeling, the MSC-Exos were resuspended in PBS after 75 min of ultracentrifugation at 100,000*g*.

### Preparation and characterization of the MSC-Exos and Exo-loaded hydrogels

The entire process, including the separation, culture, and identification of MSCs, and the collection of conditioned medium, met the stipulated quality standards. Clinical-grade MSCs were grown in 75-cm^2^ cell culture flasks at 37 °C with 5% CO_2_ in 10% FBS-treated DMEM/F12 for 48 h after seeding at a density of 1 × 10^4^ cells/cm^2^. To extract Exos, the conditioned media were collected and then centrifuged for 1 h at 4 °C at 110,000*g*. NTA and TEM were used to verify the development of Exos. The Exo markers TSG101 and CD63 were evaluated using Western blotting. For the preparation of the photoactive hydrogel complex, PEGBA (20 wt%), CMCS (3 wt%), and GelMA (20 wt%) solutions were mixed as previously described. In brief, PKH26-labeled Exos were introduced into the CMCS mixture. To evaluate the spread of PKH26-labeled Exos in the hydrogel and the process of phagocytosis by nasal mucosal epithelial cells, confocal laser scanning microscopy (ZEISS) was used.

### Swelling ratio calculation

To determine the swelling ratio, the initially weighed dry hydrogel samples (M0) were immersed in PBS at 37 °C. At specified time intervals, the samples were retrieved from PBS, and their weights were measured (Ms). The swelling ratio was calculated via the following formula: Swelling ratio = (Ms − M0)/M0.

### Evaluation of adhesion

The samples were placed on pigskin coverings for adhesion evaluation, and the skin samples were pulled and twisted to gauge the quality of the attachment.

### In vitro biological effects of Exo-loaded hydrogels

#### HNEpCs engulf PKH26-labeled exosomes

HNEpCs were seeded into 24-well plates at a density of 2 × 10^4^ per well (Corning, USA). After cell adhesion, PKH26-labeled exosomes were introduced and then incubated for 12 or 24 h. Following permeabilization with 0.3% Triton X-100 (Beyotime, China) and fixation with 4% paraformaldehyde, HNEpCs were sequentially incubated at room temperature with 4′,6-diamidino-2-phenylindole (DAPI) and phalloidin. A fluorescence microscope was used to view the cells.

#### Assays for cell proliferation

HNEpC vitality was assessed via the CCK-8 test. The cells were subjected to various treatments for 1 or 2 d, followed by incubation with CCK-8 at 37 °C. The absorbance at 450 nm was determined using a BioTech, TX, USA reader for microplates. Furthermore, live–dead experiments were performed to assess HNEpC apoptosis. Then, 0.1% (v/v) propidium iodide and 0.1% (v/v) calcein AM were used to stain the cells. Following a 30-min incubation period at 37 °C, confocal laser scanning microscopy (Leica) was used to visualize the samples. ImageJ was used to calculate the percentage of live cell fluorescence intensity to quantify cell viability.

#### Wound healing and Transwell assays

HNEpCs and fibroblasts were cultivated to approximately 100% confluence in cell culture plates measuring 35 mm. For the Transwell and wound scratch tests, the cells were deprived of serum media for 12 h before being subjected to different treatments. A sterile 200-μl pipette tip was used to create a wound gap on the monolayer. The samples were then washed with PBS and treated with various culture solutions. The migration of the cells at 0, 12, and 24 h was recorded via microscopy. For the Transwell test, 200 μl of a fibroblast suspension (5 × 10^4^/well) was placed in the chamber of a 24-well Transwell plate with an 8-μm membrane filter for the aperture (Corning, NY). The lower chamber was then filled with 500 μl of culture medium supplemented with a solution, after which it was incubated for a full day. The migratory fibroblasts were fixed with 4% paraformaldehyde, labeled with 0.5% crystal violet, and viewed under an Olympus FSX100 microscope (Japan).

#### Immunofluorescence staining

The cells or tissues were treated with 4% paraformaldehyde at 37 °C for 20 min to prepare them for immunofluorescence staining. Following an hour of permeabilization with 0.1% Triton X-100, the samples were neutralized for 30 min with 3% albumin from cow serum. The samples were then incubated with primary antibodies, such as α-SMA (1:1,000, Abcam) and integrin (1:1,000, Abcam), overnight at 4 °C. The secondary antibodies were then incubated with the samples for 1 h at 37 °C. A confocal laser scanning microscope was used for visualization.

#### Evaluation of antibacterial activity

*S. aureus* was selected as the model organism to evaluate the antibacterial activity of the hydrogels. The hydrogel (approximately 1 ml) was prepared and irradiated with UV light on a superclean table for 15 min. After autoclaving the nutrient agar medium, 1 ml of a PBS-diluted bacterial suspension of *S. aureus* (approximately 10^7^ colony-forming units/ml) was evenly smeared on agar plates via the plate-coating method. The hydrogel samples were placed lightly in the middle of the agar plates. Following overnight incubation at 37 °C, the zone of inhibition was measured to compare the antibacterial effects.

### In vivo evaluation of Exo-loaded hydrogels

#### Nasal mucosal injury surgery and wound healing evaluation

Thirty-two mature New Zealand white rabbits were split into 4 groups at random: ME-Gel (*n* = 8), hydrogel (*n* = 8), Exos (*n* = 8), and sham (*n* = 8). Following anesthesia, the nasal cavity was opened along the back of the nose, and the mucosa on the surface of the nasal septum was cut and peeled, with dimensions of approximately 15 mm × 5 mm. The injured site was then treated via a variety of techniques. Following suturing of the rabbit nose wound, intramuscular injections of penicillin and antondine were administered for 3 consecutive days after the operation.

#### In vivo biocompatibility studies

The toxicity and in vivo breakdown of the hydrogel were evaluated in New Zealand white rabbits. The hydrogels were administered subcutaneously into the dorsal side of the rabbits for 7 d to assess any potential toxicity. Important organ samples, such as those from the kidneys, liver, spleen, heart, and lungs, were collected and dyed with H&E to look for signs of material toxicity. The Nanjing Drum Tower Hospital’s Animal Investigation Ethics Committee approved all the animal experiments (approval no. 2020AE01109), which were conducted in compliance with ethical standards.

### Statistical analysis

All experiments were conducted in triplicate, and the experimental data were expressed as mean ± standard deviation. Statistical analyses were performed using Origin 2021, employing GraphPad Prism 5, one-way analysis of variance (ANOVA) to assess the significance of the results.

## Data Availability

The data that support the findings of this study are available from the corresponding author upon reasonable request.
