# Supplementary material for: Mesenchymal Stem Cell Exosome-Integrated Antibacterial Hydrogels for Nasal Mucosal Injury Treatment
Source: Research (Wash D C). 2024 Sep 9;7:0469. doi: 10.34133/research.0469 (PMC11382016; doi:10.34133/research.0469)
Supplement: Supplementary 1 — Figs. S1 to S9 [file research.0469.f1.zip › Supplementary materials.docx]

**Supplementary**

**MSC-Exosomes integrated antibacterial hydrogels for nasal mucosal injury treatment**

Min Li ^1, 2^, Rui Liu ^1^, Guopu Chen ^1^, Handong Wang ^1^, Jinglin Wang ^1,^ *, Bin Kong ^3, 4,^ *, Chenjie Yu ^1, 2,^ *

*1 Department of Otolaryngology Head and Neck Surgery, Nanjing Drum Tower Hospital, Jiangsu Provincial Key Medical Discipline, Medical School, Nanjing University, Nanjing, 210008, China*

*2 Department of Otolaryngology Head and Neck Surgery, Affiliated Nanjing Drum Tower Hospital Clinical College of Xuzhou Medical University, Nanjing, 210008, China*

*3 Guangdong Key Laboratory of Biomedical Measurements and Ultrasound Imaging, Department of Biomedical Engineering, School of Medicine, Shenzhen University, Shenzhen, Guangdong 518000, China*

*4 Department of Neurosurgery, Health Science Center, The First Affiliated Hospital of Shenzhen University, Shenzhen Second People's Hospital, Shenzhen, Guangdong 518035, China*

*E-mail: entphd@163.com; kongbin13@163.com; cw20120817@163.com


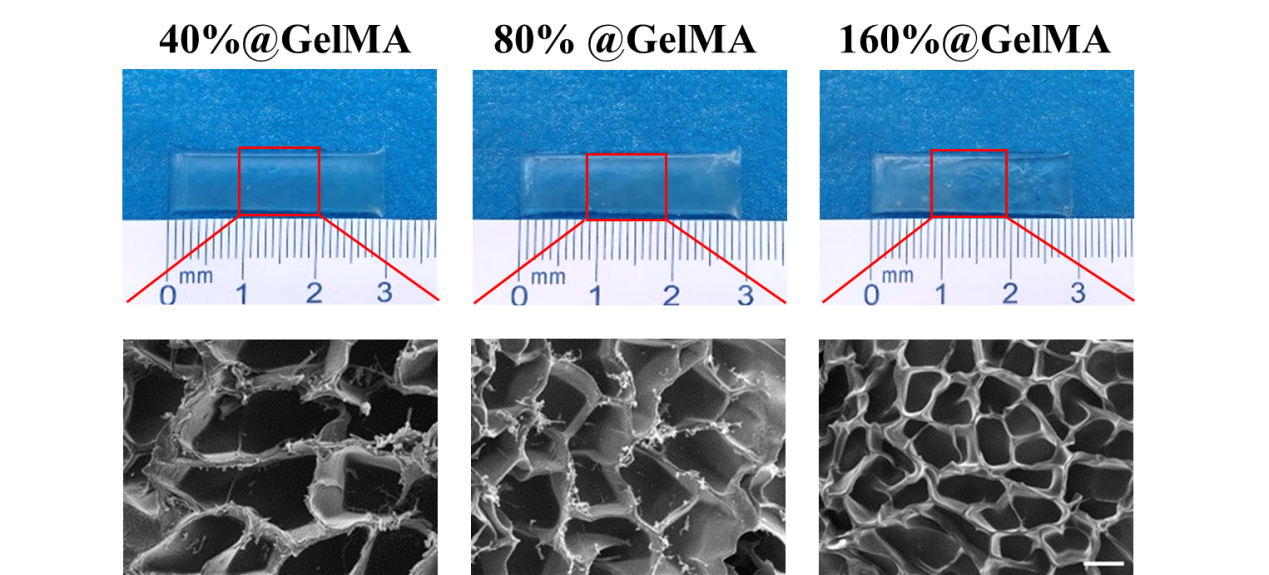


**Figure S1.** Sem structure of hydrogels with different concentrations. Scale bar = 100 μm.

**Figure S2.** Cumulative release profile of exosomes from the Hydrogel within 14 days. *p < 0.05, **p < 0.01, ***p < 0.001, ****p < 0.0001, not significant (ns).


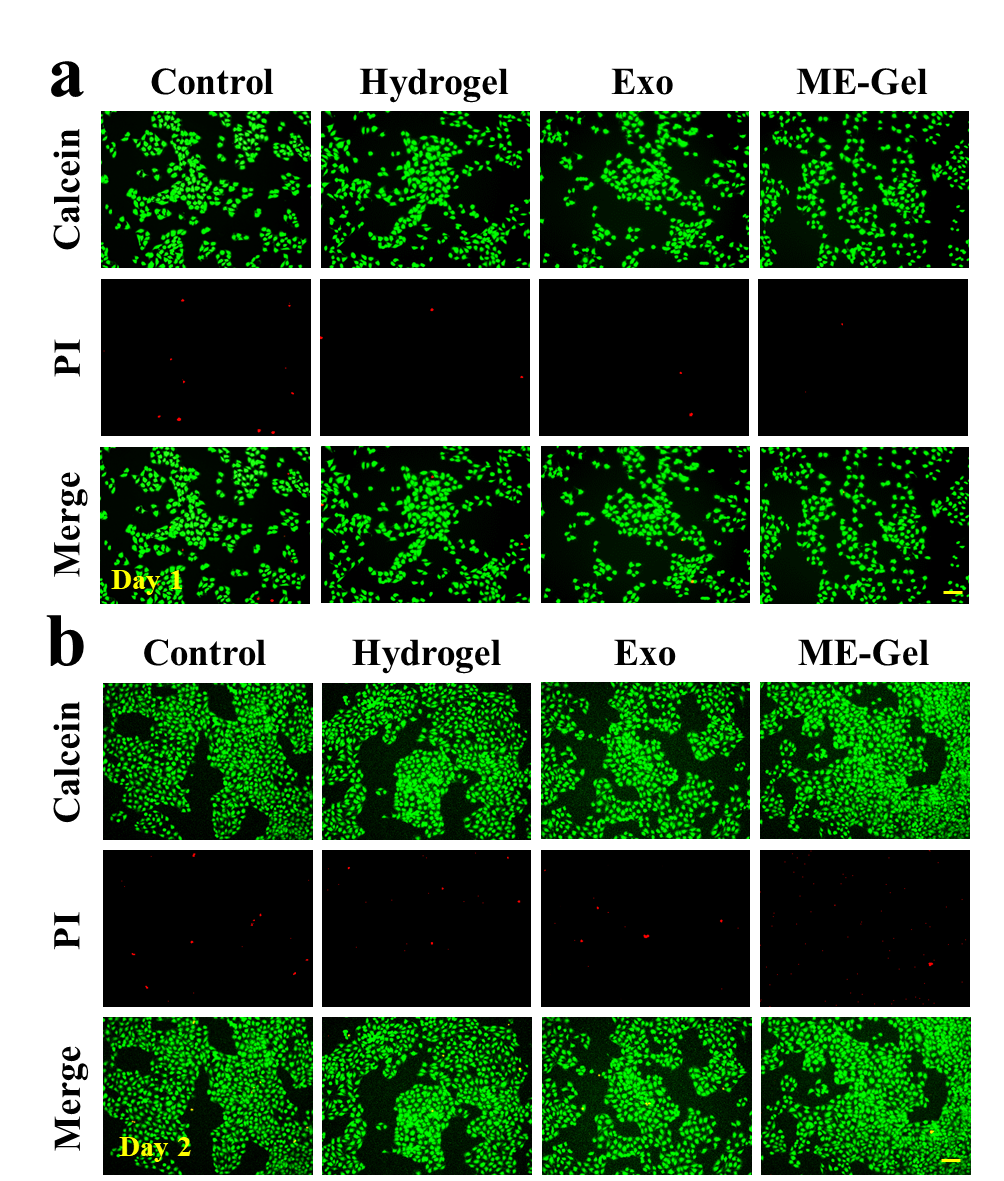


**Figure S3.** Live/Dead fluorescence staining of Nasal mucosal epithelial cells on 1 and 2 days. Green fluorescent cells labled with Calcein AM are alive and red fluorescent cells marked with propidium iodide indicate dead. Scale bar: 100 µm.


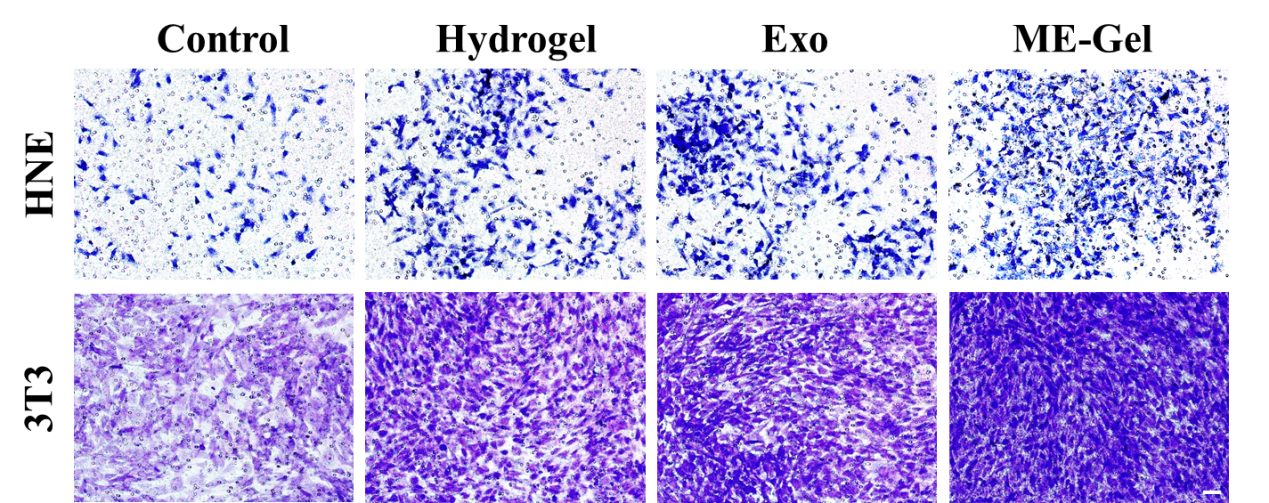


**Figure S4.** Migration ability of HNEpCs and fibroblasts under different conditions in transwell experiment. Scale bar: 100 µm.


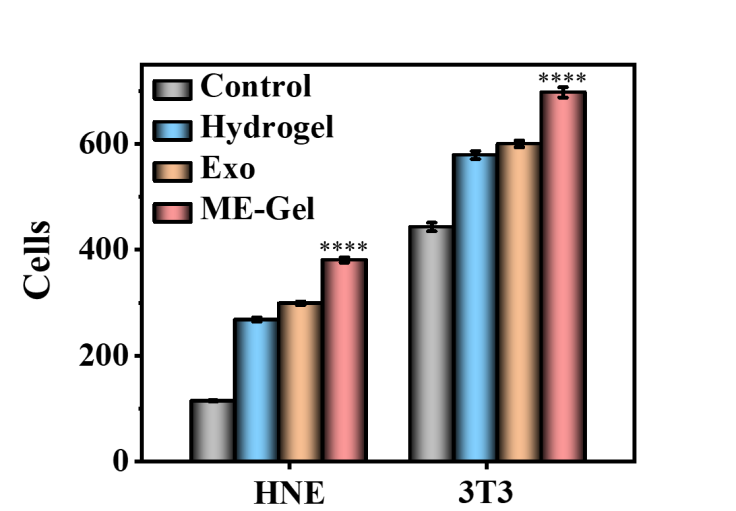


**Figure S5.** The statistical diagram of migration ability of HNEpCs and fibroblasts under different conditions in transwell experiment. *p < 0.05, **p < 0.01, ***p < 0.001, ****p < 0.0001, not significant (ns).


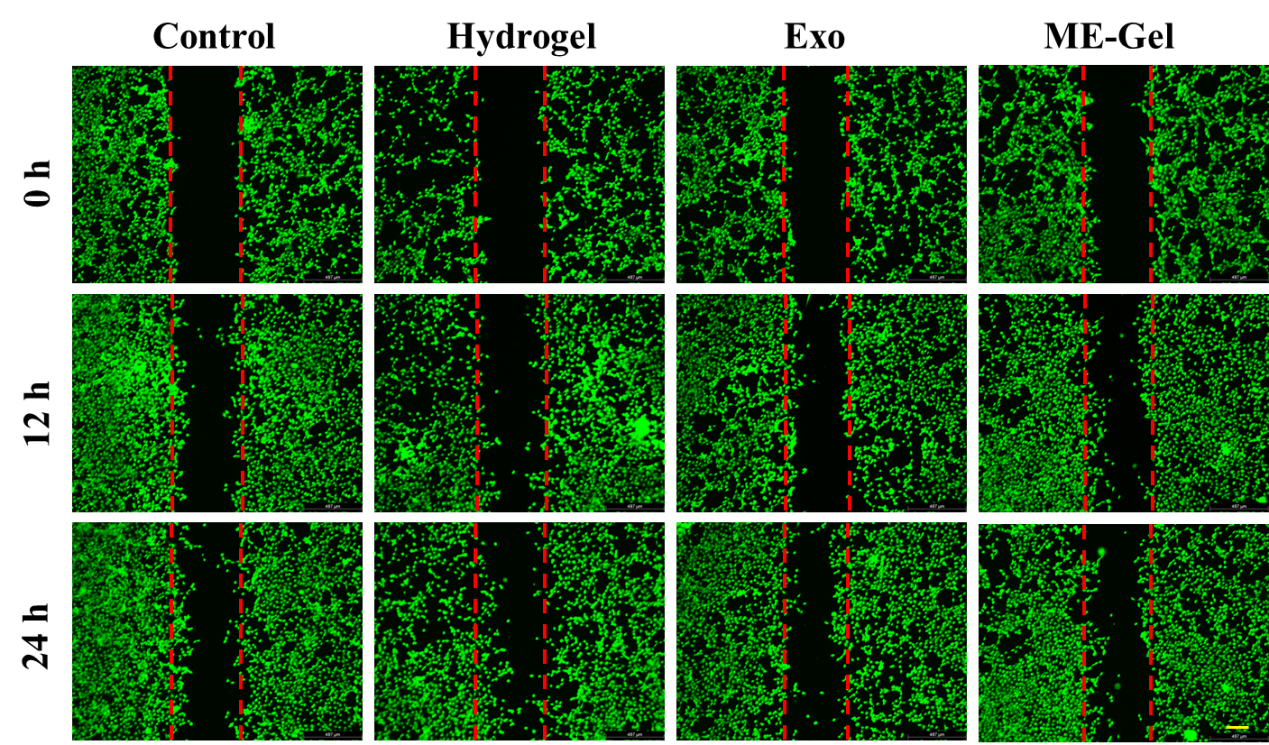


**Figure S6.** The effect of ME-Gel on fibroblasts evaluated by the scratch wound assays. The scale bar is 100 μm.


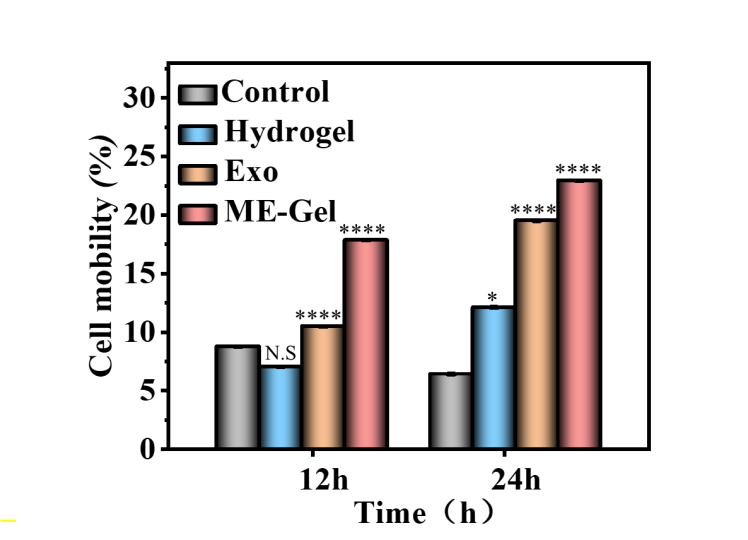


**Figure S7.** The statistical diagram of the effect of ME-Gel on fibroblasts evaluated by the scratch wound assays. *p < 0.05, **p < 0.01, ***p < 0.001, ****p < 0.0001, not significant (ns).


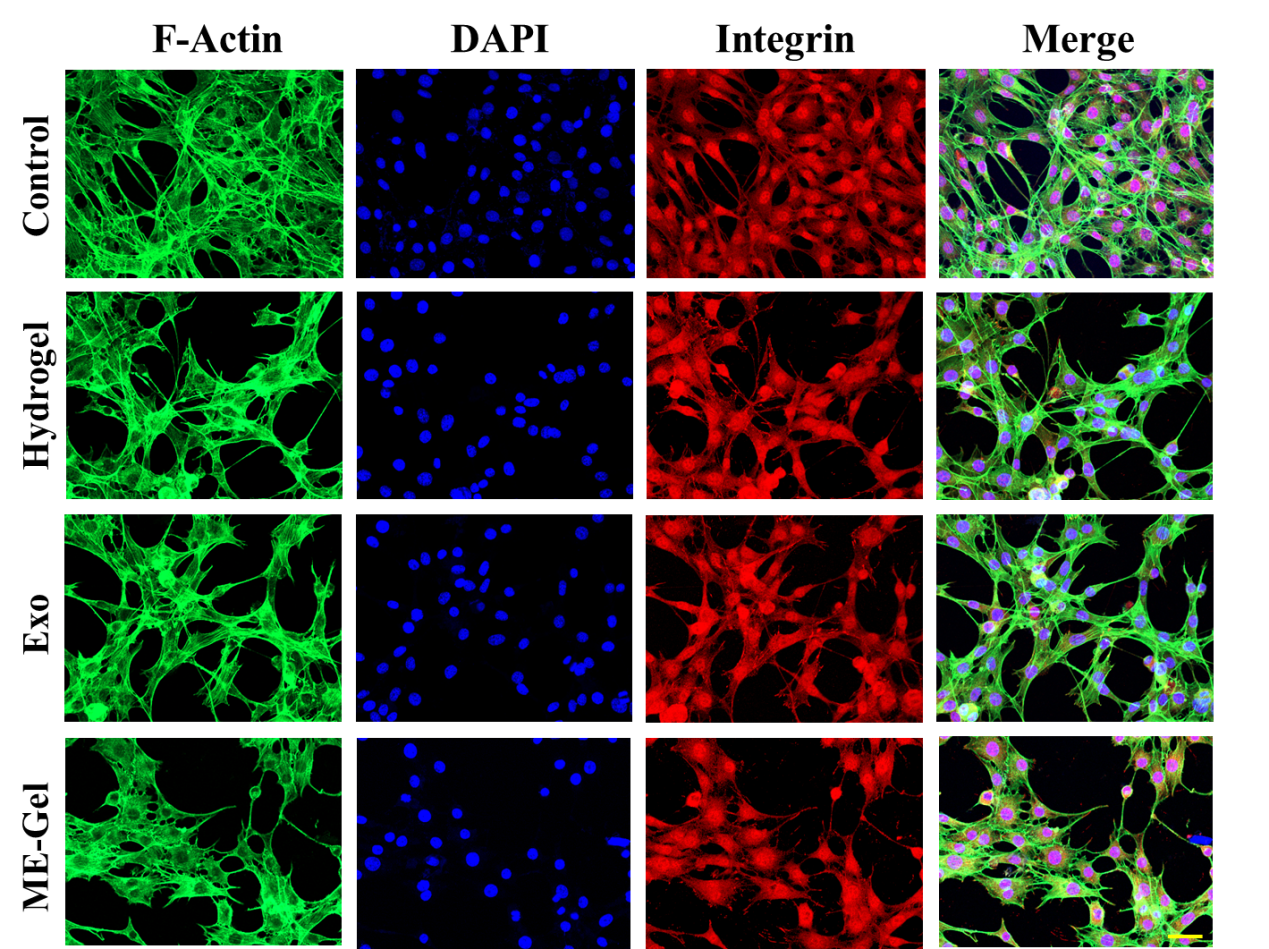


**Figure S8.** The expression of integrin (red) in fibroblasts after coculture with ME-Gel for 1 day. The scale bar is 50 μm.


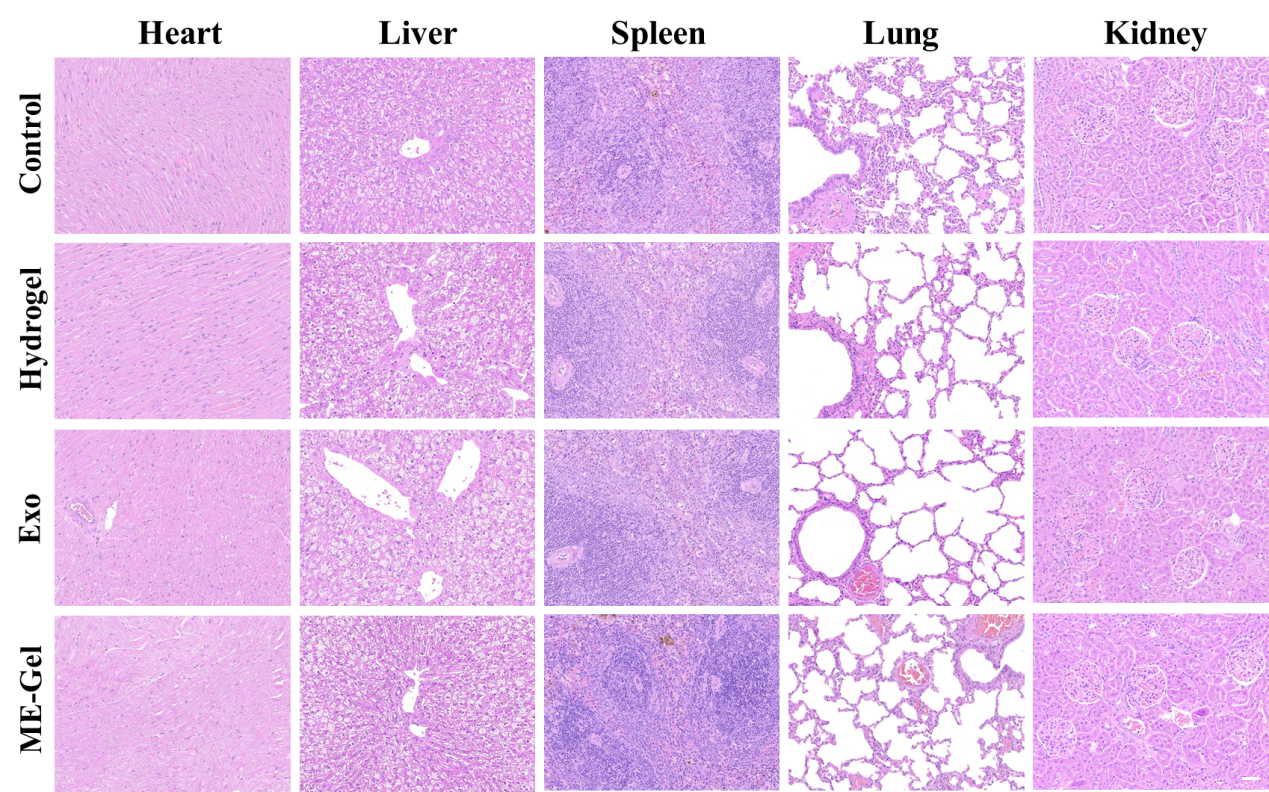


**Figure S9.** Representative picture of HE staining for main organs. Scale bar: 200 μm.
