# Supplementary figures and images for: Mesenchymal Stem Cell Exosome-Integrated Antibacterial Hydrogels for Nasal Mucosal Injury Treatment
Source: Research (Wash D C). 2024 Sep 9;7:0469. doi: 10.34133/research.0469 (PMC11382016; doi:10.34133/research.0469)

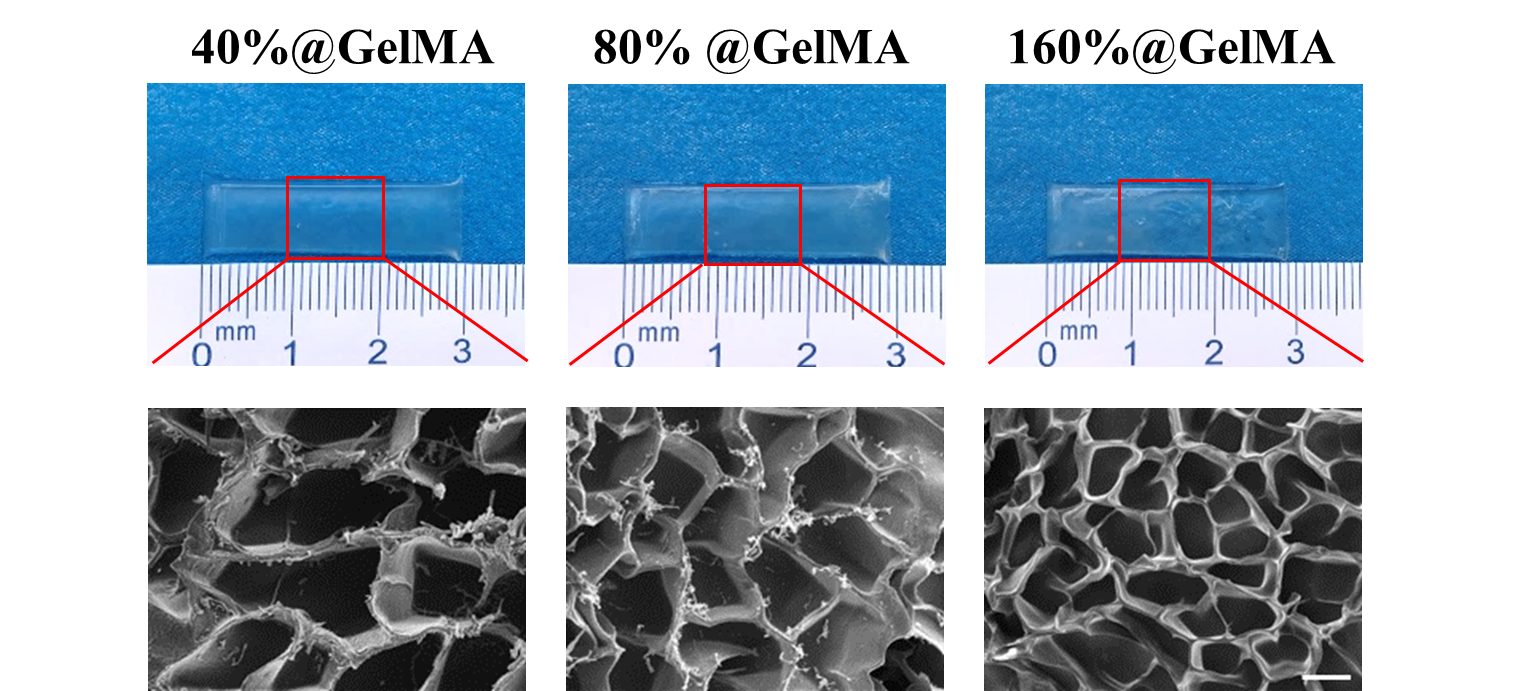

Supplement: Supplementary 1 — Figs. S1 to S9 [file research.0469.f1.zip › SI1.tif]

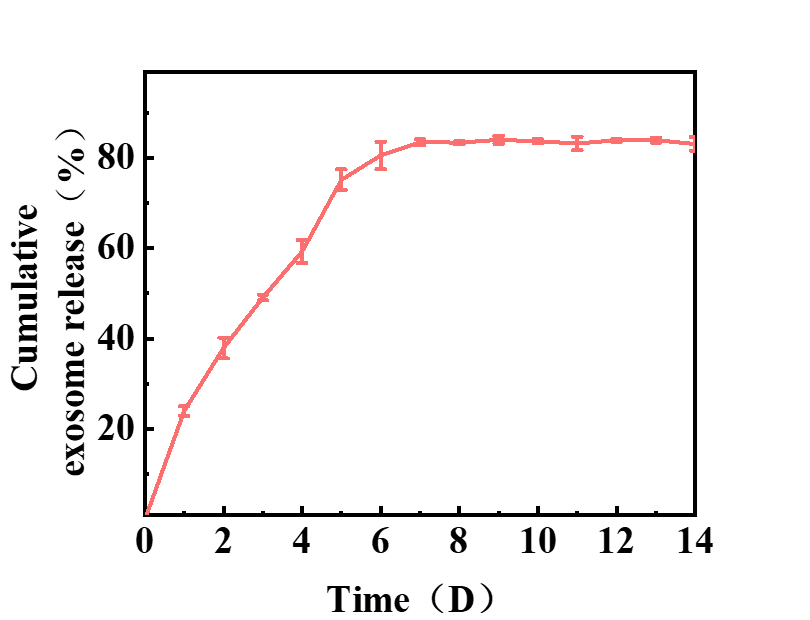

Supplement: Supplementary 1 — Figs. S1 to S9 [file research.0469.f1.zip › SI2.tif]

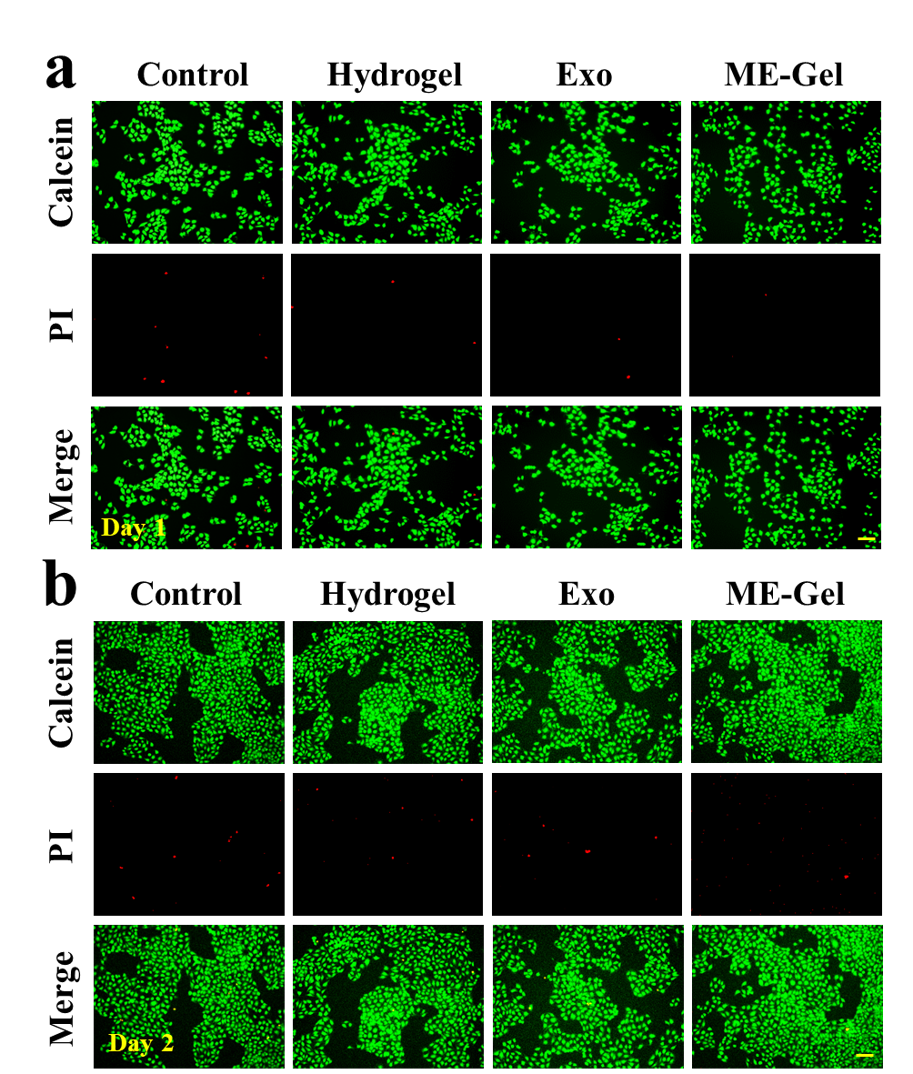

Supplement: Supplementary 1 — Figs. S1 to S9 [file research.0469.f1.zip › SI3.tif]

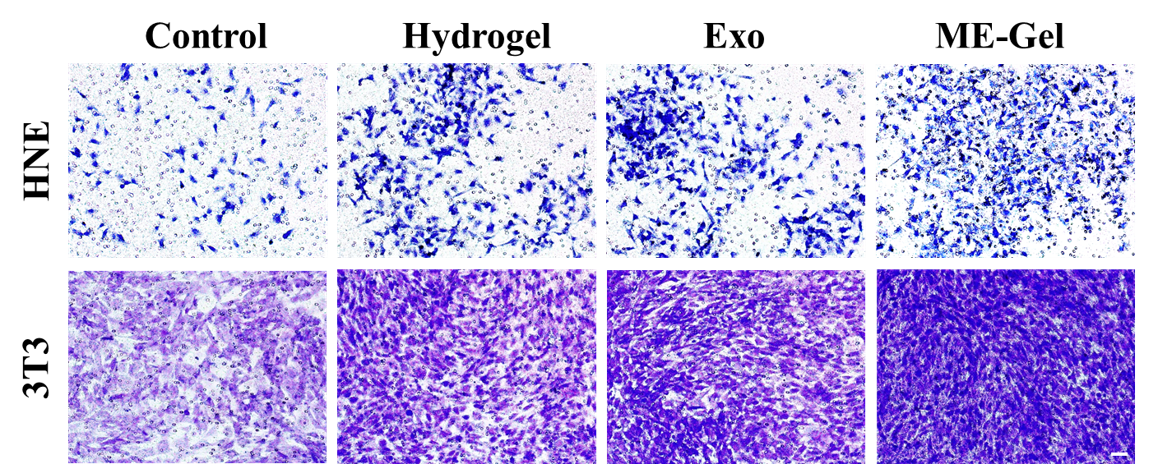

Supplement: Supplementary 1 — Figs. S1 to S9 [file research.0469.f1.zip › SI4.tif]

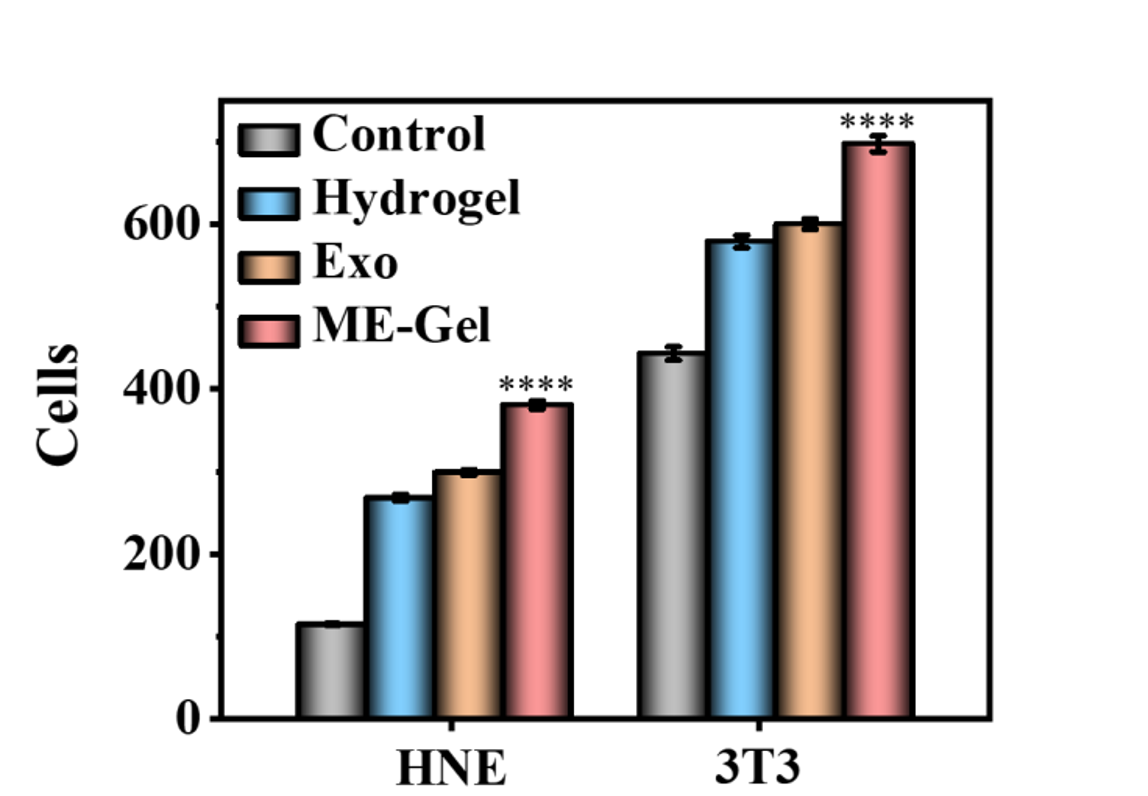

Supplement: Supplementary 1 — Figs. S1 to S9 [file research.0469.f1.zip › SI5.tif]

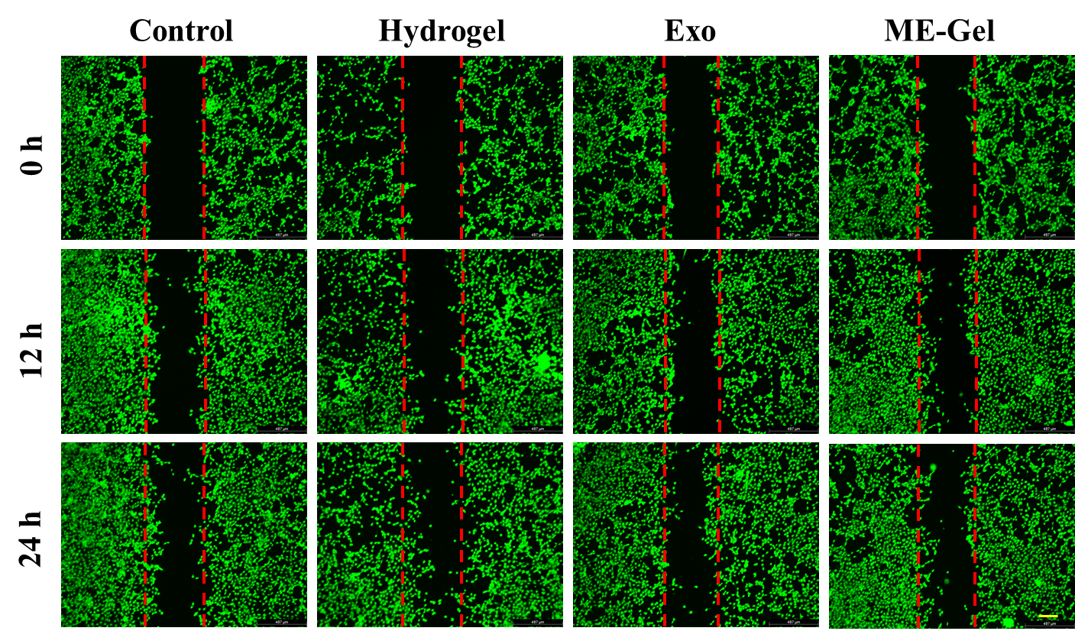

Supplement: Supplementary 1 — Figs. S1 to S9 [file research.0469.f1.zip › SI6.tif]

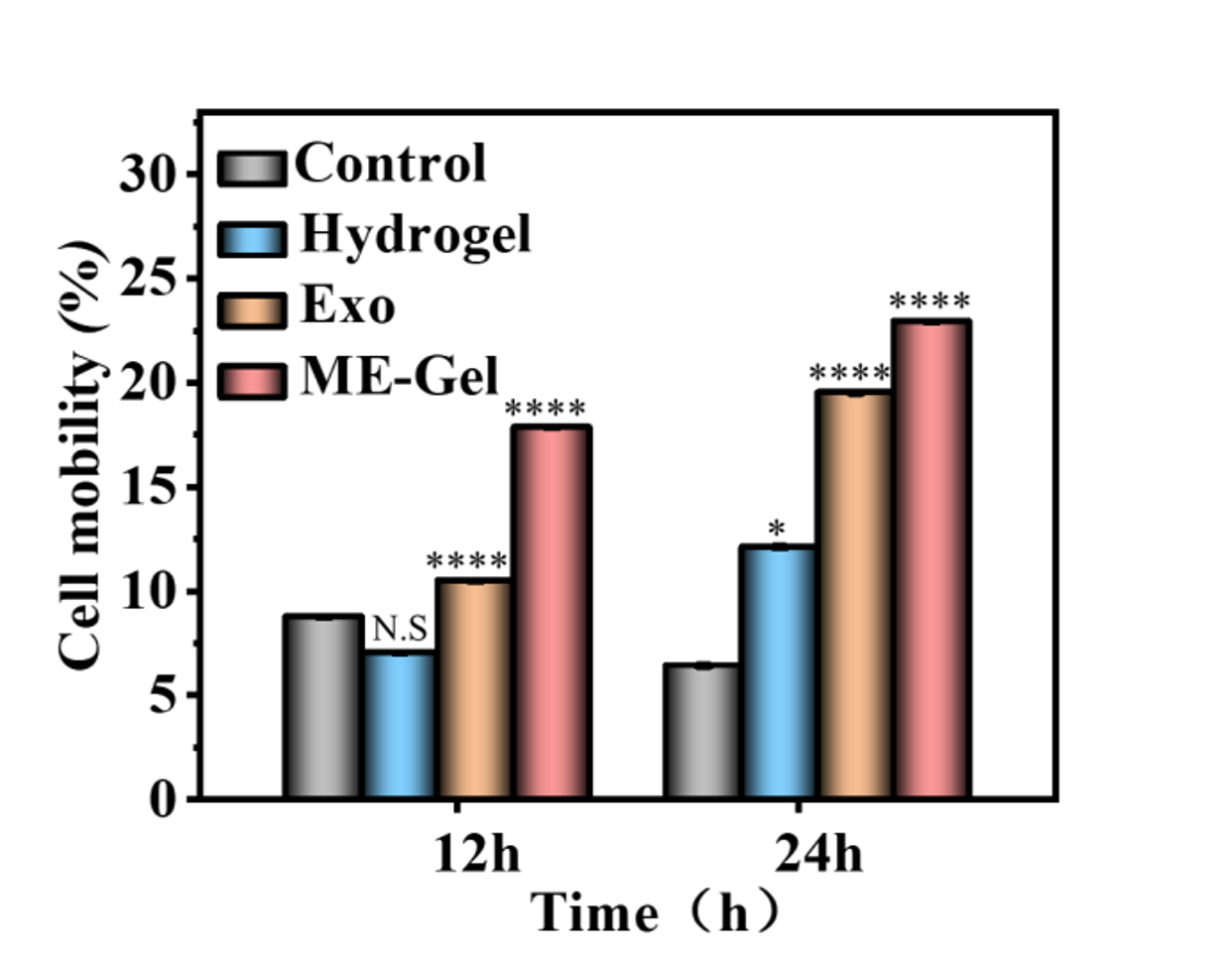

Supplement: Supplementary 1 — Figs. S1 to S9 [file research.0469.f1.zip › SI7.tif]

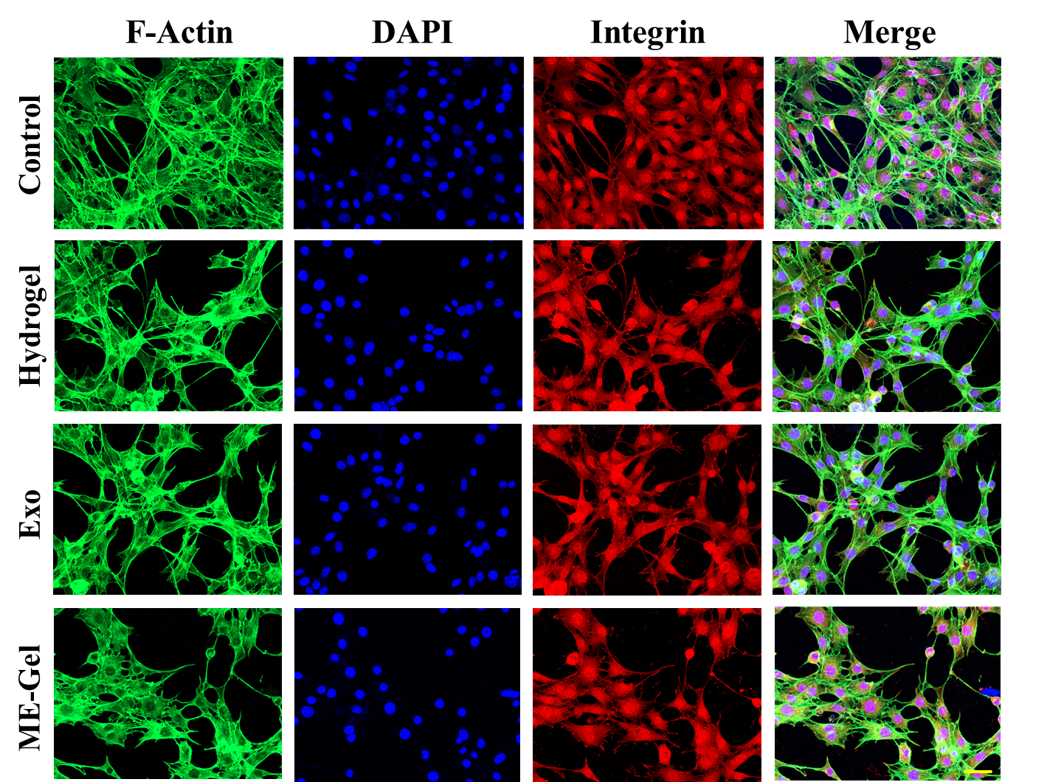

Supplement: Supplementary 1 — Figs. S1 to S9 [file research.0469.f1.zip › SI8.tif]

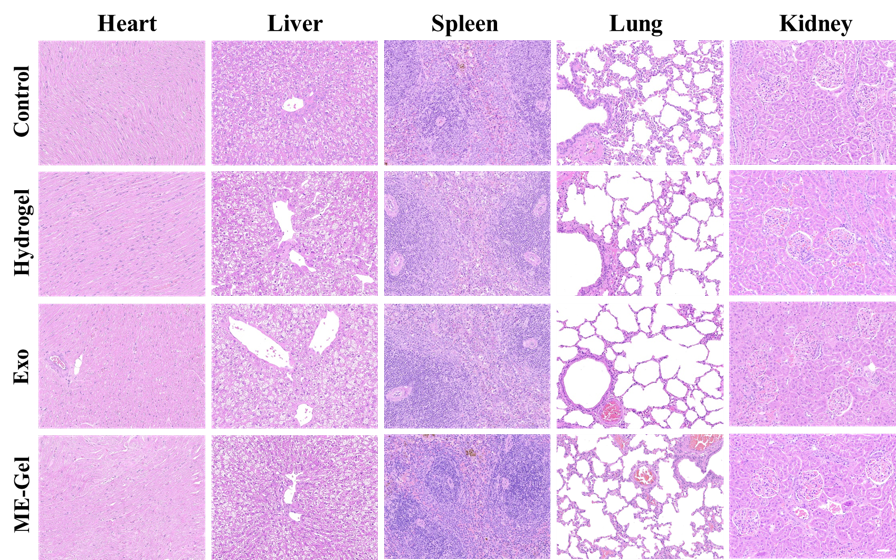

Supplement: Supplementary 1 — Figs. S1 to S9 [file research.0469.f1.zip › SI9.tif]
